# Effect of metabolically divergent pig breeds and tissues on mesenchymal stem cell expression patterns during adipogenesis

**DOI:** 10.1186/s12864-024-10308-z

**Published:** 2024-04-25

**Authors:** Siriluck Ponsuksili, Puntita Siengdee, Shuaichen Li, Wannapimol Kriangwanich, Michael Oster, Henry Reyer, Klaus Wimmers

**Affiliations:** 1 Research Institute for Farm Animal Biology (FBN), Wilhelm-Stahl-Allee 2, 18196 Dummerstorf, Germany; 2 Program in Applied Biological Sciences: Environmental Health, Chulabhorn Graduate Institute, Kamphaeng Phet 6 Road, Laksi, 10210 Bangkok, Thailand; 3 Department of Veterinary Biosciences and Public Health, Faculty of Veterinary Medicine, Chiang Mai University, 50100 Chiang Mai, Thailand; 4 Faculty of Agricultural and Environmental Sciences, University Rostock, 18059 Rostock, Germany

**Keywords:** Pig, Mesenchymal stem cells, Adipogenesis, Adipocyte

## Abstract

**Background:**

Unraveling the intricate and tightly regulated process of adipogenesis, involving coordinated activation of transcription factors and signaling pathways, is essential for addressing obesity and related metabolic disorders. The molecular pathways recruited by mesenchymal stem cells (MSCs) during adipogenesis are also dependent on the different sources of the cells and genetic backgrounds of donors, which contribute to the functional heterogeneity of the stem cells and consequently affect the developmental features and fate of the cells.

**Methods:**

In this study, the alteration of transcripts during differentiation of synovial mesenchymal stem cells (SMSCs) derived from fibrous synovium (FS) and adipose synovial tissue (FP) of two pig breeds differing in growth performance (German Landrace (DL)) and fat deposition (Angeln Saddleback (AS)) was investigated. SMSCs from both tissues and breeds were stimulated to differentiate into adipocytes in vitro and sampled at four time points (day 1, day 4, day 7 and day 14) to obtain transcriptomic data.

**Results:**

We observed numerous signaling pathways related to the cell cycle, cell division, cell migration, or cell proliferation during early stages of adipogenesis. As the differentiation process progresses, cells begin to accumulate intracellular lipid droplets and changes in gene expression patterns in particular of adipocyte-specific markers occur. PI3K-Akt signaling and metabolic pathways changed most during adipogenesis, while p53 signaling and ferroptosis were affected late in adipogenesis. When comparing MSCs from FS and FP, only a limited number of differentially expressed genes (DEGs) and enriched signaling pathways were identified. Metabolic pathways, including fat, energy or amino acid metabolism, were highly enriched in the AS breed SMSCs compared to those of the DL breed, especially at day 7 of adipogenesis, suggesting retention of the characteristic metabolic features of their original source, demonstrating donor memory in culture. In contrast, the DL SMSCs were more enriched in immune signaling pathways.

**Conclusions:**

Our study has provided important insights into the dynamics of adipogenesis and revealed metabolic shifts in SMSCs associated with different cell sources and genetic backgrounds of donors. This emphasises the critical role of metabolic and genetic factors as important indications and criteria for donor stem cell selection.

**Supplementary Information:**

The online version contains supplementary material available at 10.1186/s12864-024-10308-z.

## Background

The mesenchymal stem cells (MSCs) are multipotent progenitors cells that can differentiate into a variety of cell types, including bone cells, cartilage cells, and fat cells [1]. Adipogenesis, the differentiation of mesenchymal stem cells into adipocytes, entails activating transcription factors and signaling pathways. It comprises a two-step process: stem cell determination and preadipocyte differentiation [2–4]. Briefly, at the beginning of adipogenesis is the signaling of bone morphogenetic proteins (BMPs), which belong to the transforming growth factor β (TGF-β) superfamily, a family of proteins that play a role in the conversion of pluripotent stem cells into the adipocyte lineage [5]. Then it is often portrayed as a cascade of regulatory events, the first wave involving CCAAT/enhancer-binding protein β (C/EBPβ) and C/EBPδ and sterol-regulatory element binding protein 1 (SREBP1), and these transcription factors being involved in the activation of the second wave, which includes C/EBPα and PPARγ, which coordinately activate the transcription of genes that give rise to the adipocyte phenotype [4].

Pigs, due to their physiological similarities to humans, serve as valuable cellular models for advancing stem cell therapies, regenerative medicine, and transplantation [6, 7]. Porcine MSCs are utilized as large animal models in regenerative medicine, preclinical studies, and transplantation for both human and veterinary applications, benefiting the livestock industry [7–9]. Comprehensive understanding of molecular changes during MSC transition from self-renewal to differentiation, particularly in pig adipogenesis, and related technologies, contributes to diverse experimental research.

Various source of tissue for derived MSCs influence functional properties of the cells was reported [10–13]. Bone marrow and subcutaneous adipose tissue are the most familiar sources for MSCs derivative used in most stem cell studies, whereas synovium or infrapatellar fat pad tissues have been considered as promising MSCs sources [14–16]. In addition to the MSCs derived from synovium and infrapatellar fat pad have demonstrated higher proliferation capacity [15, 17]. Moreover, MSCs from fibrous synovium release a greater number of MSCs than adipose synovium [18]. Our prior study demonstrated that SMSCs from fibrous synovium and adipose synovial tissue exhibit similar cell morphologies and immunophenotypes but distinct molecular features [12].

The genetic background of donor MSCs also plays an important role in adipogenesis. Previous studies have shown that pre-adipocytes proliferate more rapidly in fat pigs than in lean pigs [19]. In addition, several studies demonstrated that bone marrow stem cell (BMSCs) from mouse fed a high-fat diet, increased reactive oxygen species (ROS) production [20] or that obese donors lead to undesirable proliferation, differentiation, and self-renewal of BMSCs [21]. Our earlier study on undifferentiated synovial mesenchymal stem cells (SMSCs) found breed-specific transcript and signaling pathway variations related to traits like lean stature or obesity in the cell donors [12]. Comparing adipose and bone marrow MSCs during adipogenic differentiation revealed genes well-represented across various functional categories of adipogenesis [22, 23]. Numerous studies have reported molecular changes in synovial MSCs during differentiation into adipocytes [24–26].

In this study, we gain further insight into the transcript changes during differentiation of synovial MSCs derived from fibrous synovium and adipose synovial tissue sources of two pig breeds differing in growth performance (German Landrace (DL)) and fat deposition (Angeln Saddleback (AS)). SMSCs derived from both tissues and breeds were stimulated to differentiate into adipocytes in vitro. Adipocytes were harvested at four time points (the differentiation period at day 1, day 4, day7 and day 14) to generate transcriptomic data. In addition, we compare undifferentiated cell with differentiated cells along the process to gain a better understanding of molecular pathways. Therefore, the aim of this study was to identify genes and signaling pathways that are involved in the initiation of adipogenic differentiation of SMSCs from two different synovial tissues, two breeds, and that subsequently vary between undifferentiated and differentiated cells at four different time points along the developmental process to gain a better understanding of the molecular pathways.

## Results

### Characterization of porcine SMSCs during adipogenic differentiation

**Fig. 1:**
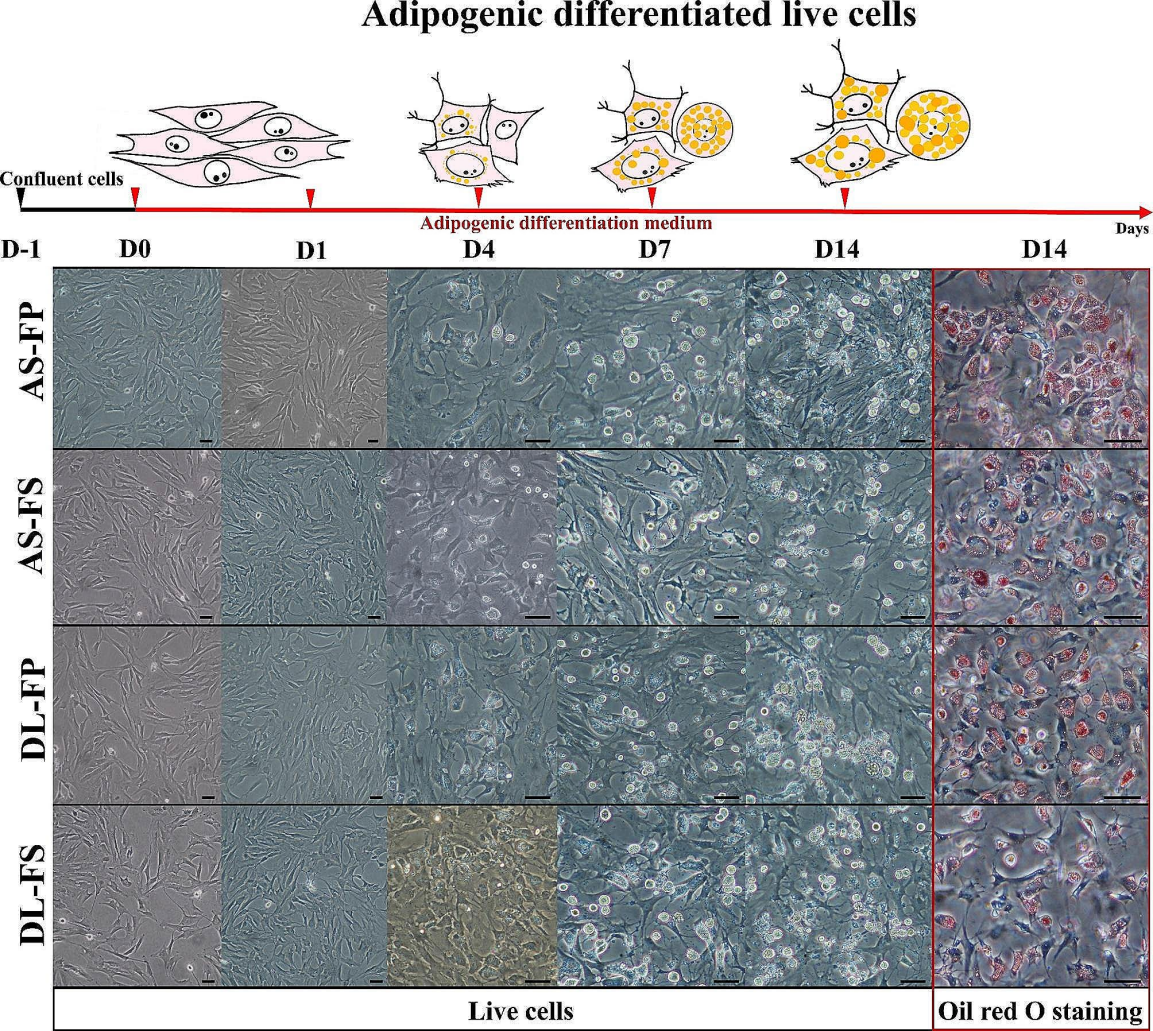
Adipogenic differentiation of mesenchymal stem cells derived from different synovial tissue sources (fibrous synovium (FS) and adipose synovial tissue (FP)) of German Landrace (DL) and Angeln Saddleback (AS) breeds. Morphological changes of live cells after plating at D0 and the differentiated cells at day 1 (D1), day 4 (D4), day 7 (D7) and day 14 (D14) were presented. The lipid-droplet formation of adipocytes was observable at 4 days, and increased over time under adipogenic differentiation after culturing in an adipogenic differentiation medium. The cytoplasmic lipid-droplets deposition was observed by Oil Red-O staining on day 14 and visualized under a phase-contrast microscope (red with a clear background). Scale bars:100 μm

The microscopic appearance of cells derived from the FS and FP tissue samples of both breeds, AS and DL, at four time points up to day 14 of cultivation under differentiation conditions are shown in Fig. 1. From day 4, lipid droplets were observed under the phase contrast microscope. An increase in lipid droplets on day 7 and even more clearly on day 14 was observed by positive red lipid droplet staining with Oil Red-O. In the control group where SMSCs were cultured in Complete Culture Medium (CCM), no lipid droplets were observed at any of the designated time points. Interestingly, no discernible differences were observed among different breeds and tissue sources in terms of these morphological characteristics, despite their differences in lipid metabolism.

### Transcriptional changes between adipogenic induction and non-inducted control groups

**Fig. 2:**
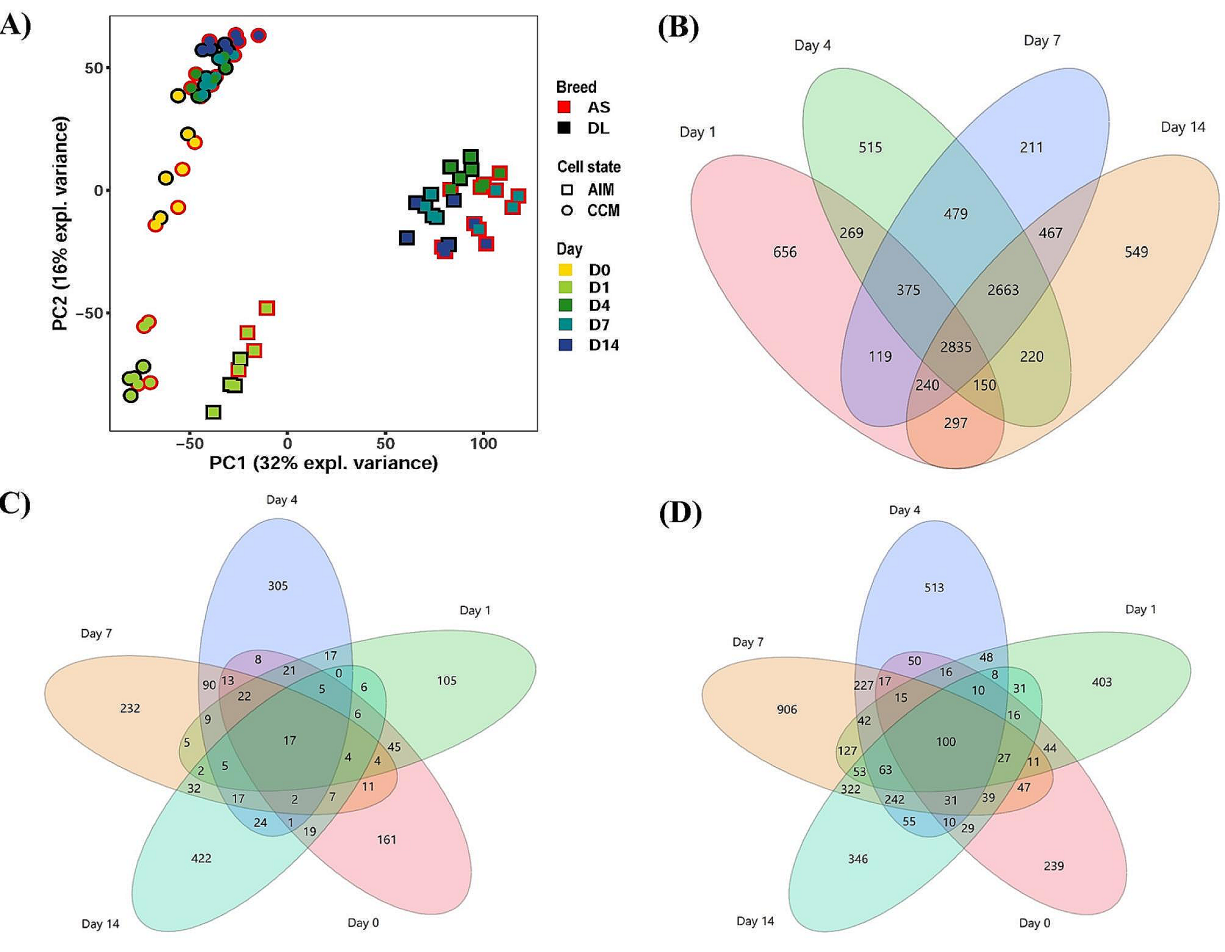
PCA plot demonstrates all microarrays used (72 samples). Samples were colored based on breeds, day of culture and symbols as cell state (**a**). Venn diagrams of the number of differentially expressed transcripts at each time point when comparing (**b**) between adipogenic induction medium (AIM) groups and complete culture medium (CCM) groups on day 1, 4, 7 and 14; (**c**) between porcine synovial mesenchymal stem cells (SMSCs) obtained from both tissue sources (fibrous synovium and adipose synovial tissue) on day 0, 1, 4, 7 and 14; (**d**) between porcine SMSCs obtained from German Landrace (DL) and Angeln Saddleback (AS) on day 0, 1, 7, 14 and 21

A total of 13,287 probe sets that met the quality control criteria were used for further investigations. Principal component analysis (PCA) was conducted using data from 13,287 probes sets encompassing all 72 samples. Notably, the PCA revealed a more pronounced segregation between cell states, particularly between the AIM groups (representing Adipogenic Induction Medium) and SMSCs cultivated in CCM (Complete Culture Medium), as illustrated in Fig. 2A, compared to the distinction between different breeds.

To investigate regulation during adipogenesis of SMSCs, we compared differentiated SMSCs grown in AIM with SMSCs grown in CCM at the same time points on day 1, day 4, day 7 and day 14, respectively (Additional file 1). Additional file 1 shows all details of the analysis with lsmean, fold change and adjusted *P*-value (FDR < 1%) with color coding of all differentially expressed genes (DEGs) between AIM groups compared to undifferentiated (CCM groups). The total number of DEGs that were up- or downregulated with an FDR < 0.01 is shown in Table 1, and the overlap number of these DEGs is shown in Fig. 2B. The number of DEGs between AIM groups and CCM groups increased from day 1 and remained constant from day 4 to day 14. There were a higher number of genes with higher abundance in CCM groups than in AIM groups. Moreover, 656, 515, 211 and 549 of these transcripts were differentially expressed between AIM groups and CCM groups exclusively on day 1, day 4, day 7 or day 14, respectively. 2835 genes were differentially expressed between AIM groups and CCM groups across both breeds at each of the four time points (Fig. 2B).

**Table 1:** Number of differentially expressed genes between adipogenesis-induced cells (AIM groups) and cells maintained under control conditions (CCM groups) from comparison within each time point (day 1, 4, 7, 14) at FDR < 0.01

	Overall	upregulated in AIM groups	upregulated in CCM groups
Day 1	4941	1928	3013
Day 4	7506	2852	4654
Day 7	7389	2915	4474
Day 14	7421	3128	4293

**Fig. 3:**
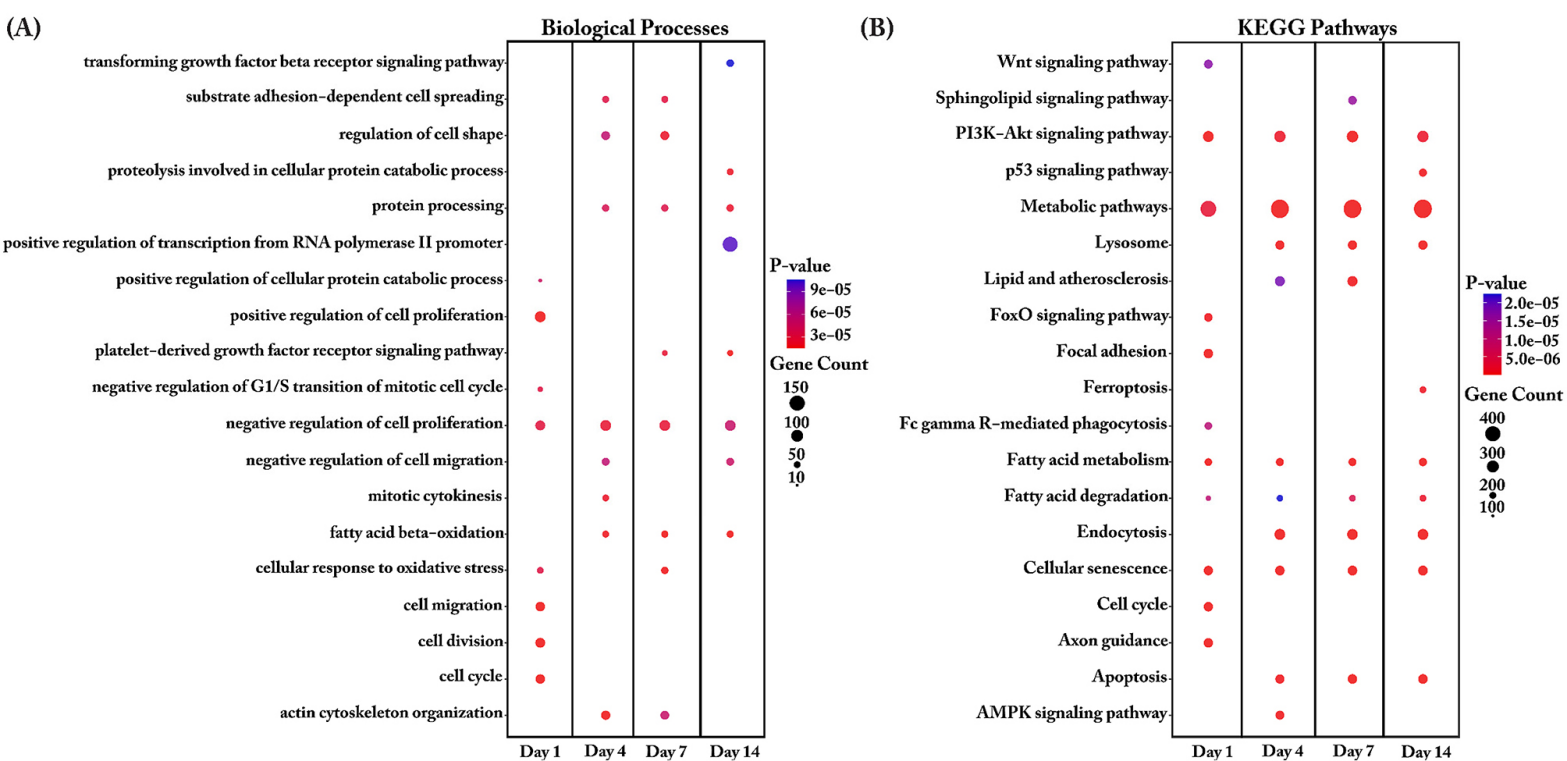
Biological Process (**a**) and KEGG pathway (**b**) enrichment analysis of differentially expressed genes between complete culture medium (CCM) groups and adipogenic induction medium (AIM) groups. The dot size represents the number of genes involved in each biological process or KEGG pathways, while the dot’s color indicates the *p-value*

The DEGs transcripts were further submitted to functional analysis for both biological process and KEGG pathway analysis. Most changes in transcript abundances at day 1 were found in categories cell cycle, cell division, cell migration or cell proliferation (Fig. 3A). On day 4 the changes in transcripts were found enriched particular in regulation of cell shape, protein process, fatty acid beta-oxidation. The biological process such as protein process and fatty acid beta-oxidation were enriched until day 14 when comparing SMSCs grown in AIM or control (CCM) medium. DEGs enriched in negative regulation of cell proliferation were found at all- time points. In the analysis of KEGG pathways (Fig. 3B), we found PI3K-Akt signaling pathways, metabolic pathways, Fatty acid metabolism, Fatty acid degradation and Cellular senescence enriched across all time points. Interesting after four days of induction, the pathways category Lysosome, Endocytosis and Apoptosis were enriched. Changes of transcripts enriched in p53 signaling pathways and ferroptosis were found on the late time point (day 14).

Upon further annotation of DEGs with up- or down-regulated at each time point during adipogenesis, we found that significant pathways of the up-regulated transcripts in the early stage (day 1 and 4) enriched in many pathways including cell cycle, DNA replication and alcoholism. Whereas at day 7 and day 14 of the up-regulated transcripts were enriched in PPAR signaling, AMPK signaling, fatty acid metabolism, regulation of lipolysis in adipocytes, unsaturated fatty acid biosynthesis, glycerolipid metabolism, adipocytokine signaling, peroxisome, steroid biosynthesis, and pyruvate metabolism.

### Comparison of the transcriptional profile of SMSCs derived from FP and FS

Depending on the origin of the tissues (FP or FS), a total of 1,617 DEGs were found at a threshold of 1% FDR in at least one of the comparisons on days 0, 1, 4, 7 and 14 (Additional file 2). The number of DEGs between FP and FS was slightly lower on day 1 than on day 0 and higher again on the late days. The most significant difference between FP and FS was observed on day 14. In addition, 161, 105, 305, 232 and 422 transcripts were found to be exclusively differentially expressed between FP and FS derived cells on day 0, day 1, day 4, day 7 or day 14, respectively (Fig. 2C). In total, 17 genes were differentially expressed depending on the origin of the tissue (FP or FS) (Fig. 2C).

**Table 2:** The number of differentially expressed genes between porcine SMSCs derived from FP or from FS from the comparisons within each time point (day 0, 1, 4, 7, 14) at FDR < 0.01

	Overall	Upregulation in FP	Upregulation in in FS	
Day 0	346	188	158	
Day 1	273	126	147	
Day 4	556	227	329	
Day 7	472	251	221	
Day 14	569	225	344	

**Fig. 4:**
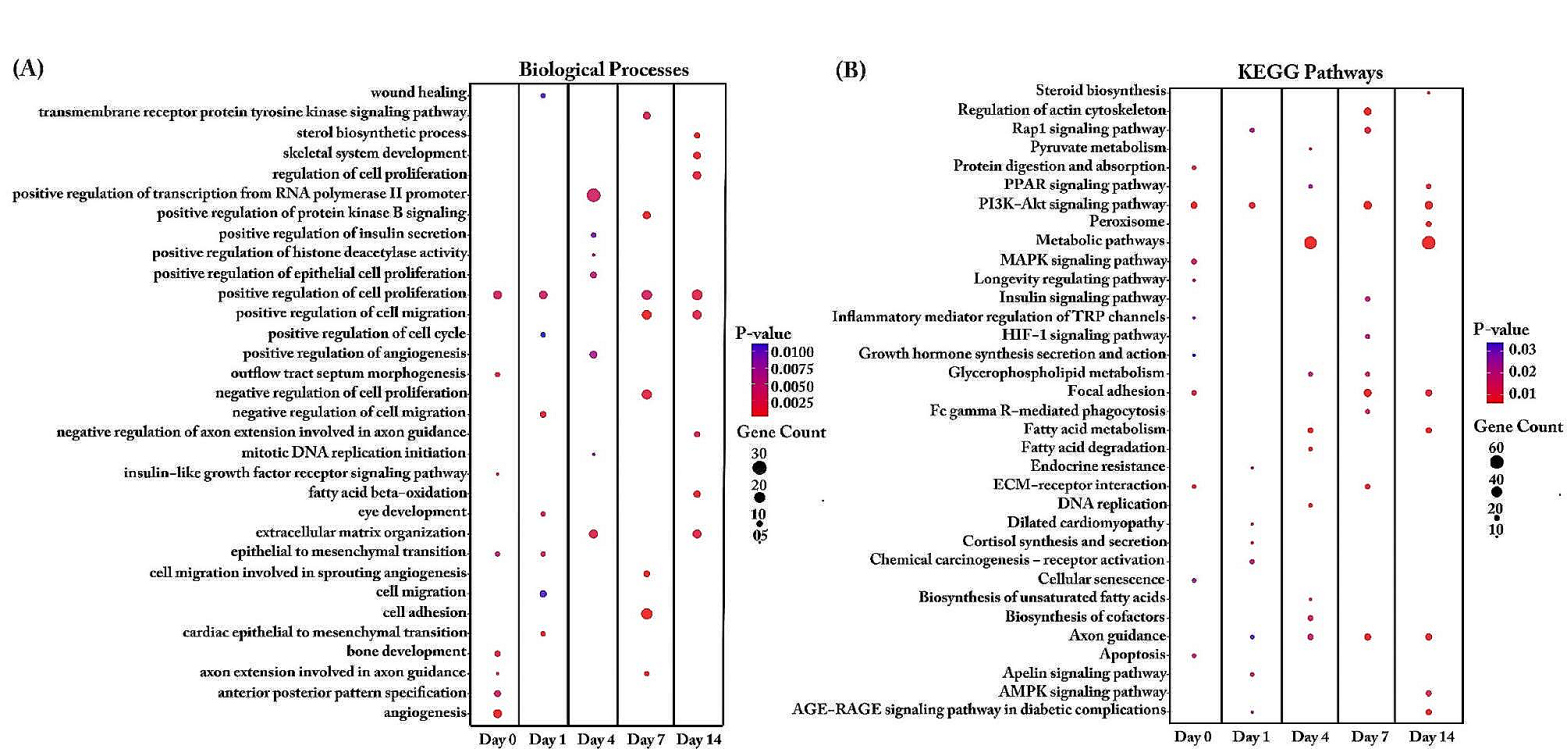
Biological Process (**A**) and KEGG pathway (**B**) enrichment analysis for differentially expressed genes of synovial mesenchymal stem cells (SMSCs) transcripts derived from fibrous synovium (FS) and adipose synovial (FP) tissue. The dot size represents the number of genes involved in each biological process or KEGG pathways, while the dot’s color indicates the *p-value*

With an overall low number of DEGs when comparing SMSCs derived from FP and FS, most biological changes were observed at day 4 to day 14. DEGs enriched in biological processes generally belong to cell biological processes such as proliferation or migration. Specific biological processes such as sterol biosynthesis or beta-oxidation of fatty acids occur at a late time point (day 14) (Fig. 4A). When analyzing KEGG signaling pathways, we found that PI3K-Akt signaling and axon guidance were enriched on most days of the comparison, while metabolic pathways were enriched on day 4 and day 14. Lipid metabolism, including glycerophospholipid, fatty acid metabolism or fatty acid degradation, was enriched on day 4 or day 7 (Fig. 4B).

### Comparison of the transcriptional profile of SMSCs derived from AS and DL

When comparing SMSCs derived from DL and AS breeds, the number of DEGs increased from day 1 (1014) to day 7 (2269) and subsequently dropped at day 14 (1382) (Table 3). The details of all DEGs before (day 0) and during adipogenesis of AS-derived SMSCs compared with DL-derived SMSCs at each time point are shown in Additional file 3. Interestingly, most upregulated genes in AS were found at day 4 (1062), whereas most upregulated genes in DL were identified at day 7 (1678) (Table 3). Moreover, 239, 403, 513, 906, and 346 of these transcripts were exclusively different between breeds at day 0, day 1, day 4, day 7, and day 14, respectively (Fig. 2D). In total, 100 transcripts were differentially expressed depending on breeds (AS or DL) across all five time points (Fig. 2D).

**Table 3:** The number of differentially expressed genes between porcine SMSCs derived from AS or from DL from the comparisons within each time point (day 0, 1, 4, 7, 14) at FDR < 0.01

	Overall	Upregulation in AS	Upregulation in DL
Day 0	701	263	438
Day 1	1014	352	662
Day 4	1447	1062	385
Day 7	2269	591	1678
Day 14	1382	494	888

**Fig. 5:**
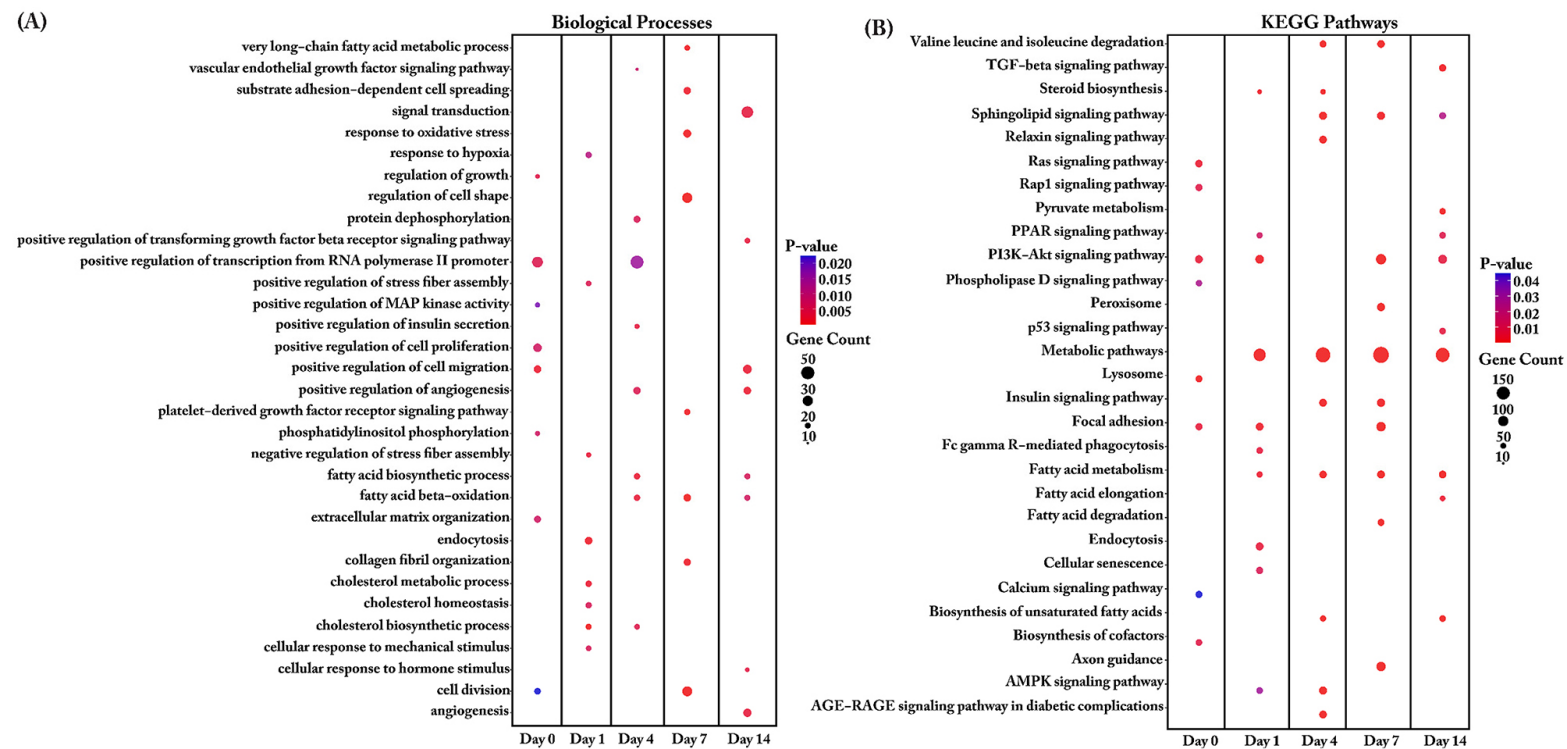
Biological Process (**A**) and KEGG pathway (**B**) enrichment analysis for differentially expressed genes of synovial mesenchymal stem cells (SMSCs) transcripts derived from German Landrace (DL) and Angeln Saddleback (AS). The dot size represents the number of genes involved in each biological process or KEGG pathways, while the dot’s color indicates the *p-value*

Biological processes in the pre-induction period were enriched in cell proliferation, cell division, cell migration, cell growth, and extracellular matrix organization (Fig. 5A). During early adipogenesis at day 1, many stress processes were enriched, including the response to hypoxia, positive/negative regulation of stress fiber assembly, cellular response to mechanical stimuli, and endocytosis. During this period, DEGs between DL and AS also enriched in the cholesterol metabolic process, including homeostasis and biosynthetic process. DEGs enriched in beta-oxidation of fatty acids were found from day 4 to day 14. At day 4, DEGs were also enriched in fatty acid biosynthesis, cholesterol biosynthesis, protein dephosphorylation, and positive regulation of angiogenesis, whereas at day 7, more changes were observed in the regulation of cell shape, cell division, and oxidative stress response. Strong enrichment in signal transduction and angiogenesis was observed on day 14. In KEGG enrichment analysis (Fig. 5B), metabolic pathways and fatty acid metabolism were observed during adipogenesis from day 1 to day 14. Certain metabolic pathways, including steroid biosynthesis, sphingolipid signaling, relaxin signaling, insulin signaling, AMPK signaling, and AGE-RAGE signaling in diabetic complications, were enriched at day 4. It is intriguing to note that, axon guidance, peroxisome, and fatty acid degradation were enriched only at day 7, whereas TGF-beta signaling, pyruvate metabolism, p53 signaling, and fatty acid elongation appeared only at day 14.

**Fig. 6:**
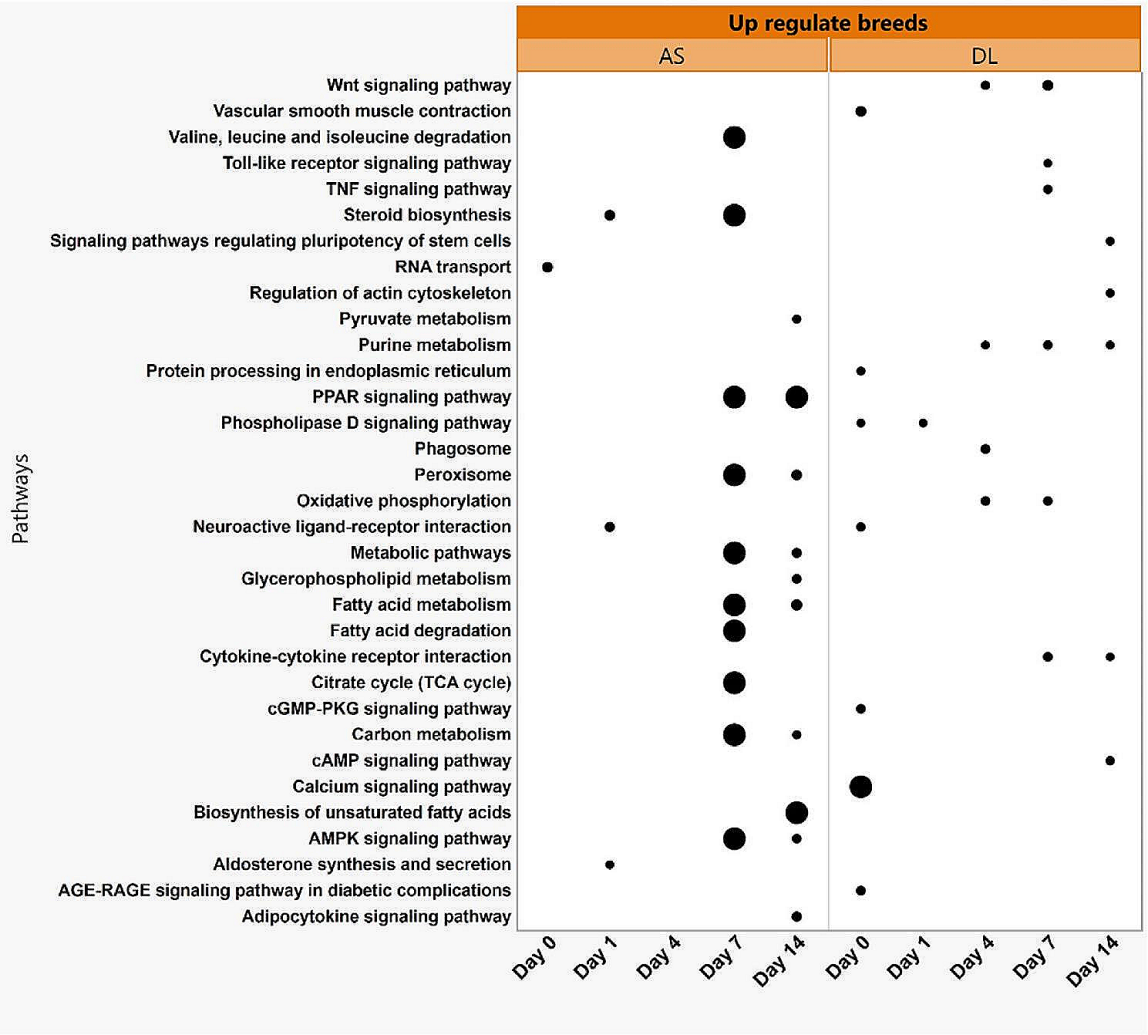
Biological processes and pathways enriched in the upregulated transcripts derived from Angeln Saddleback (AS) and German Landrace (DL) at each time points of non-adipogenesis (day 0) and during adipogenesis (day1-day14). The dot size represents the significance levels (*p* < 0.05)

A separate examination of the DEGs upregulated in each of the two breeds AS and DL at the time points day 0, day 1 and day 4 provides further insights into the breed-specific regulation of the developmental processes (Fig. 6). As shown in Table 3, most DEGs were found at day 7, with only 591 transcripts upregulated in AS, while 1678 transcripts were upregulated in DL. These upregulated transcripts in AS were enriched at a threshold of FDR < 0.05 in the metabolic pathways of fatty acid metabolism, carbon metabolism, valine, leucine and isoleucine degradation, peroxisomes, AMPK signaling, steroid biosynthesis, fatty acid degradation, citrate cycle and amino acid biosynthesis (Fig. 6). While the upregulated transcripts in DL group of the same day (day 7), greater enrichment of Wnt signaling, interaction between cytokine and cytokine receptors, oxidative phosphorylation, purine metabolism, TNF signaling and Toll-like receptor signaling was evident (*p-value* < 0.05). At day 14, the up-regulated transcripts of the AS groups showed enrichment in the metabolic pathways, PPAR signaling pathway, fatty acid metabolism and peroxisomes. While the upregulated transcripts of the DL breeds were more enriched in cytokine-cytokine receptor interaction, purine metabolism, actin cytoskeleton regulation, stem cell pluripotency regulation pathways and cAMP signaling pathway (Fig. 6).

**Fig. 7:**
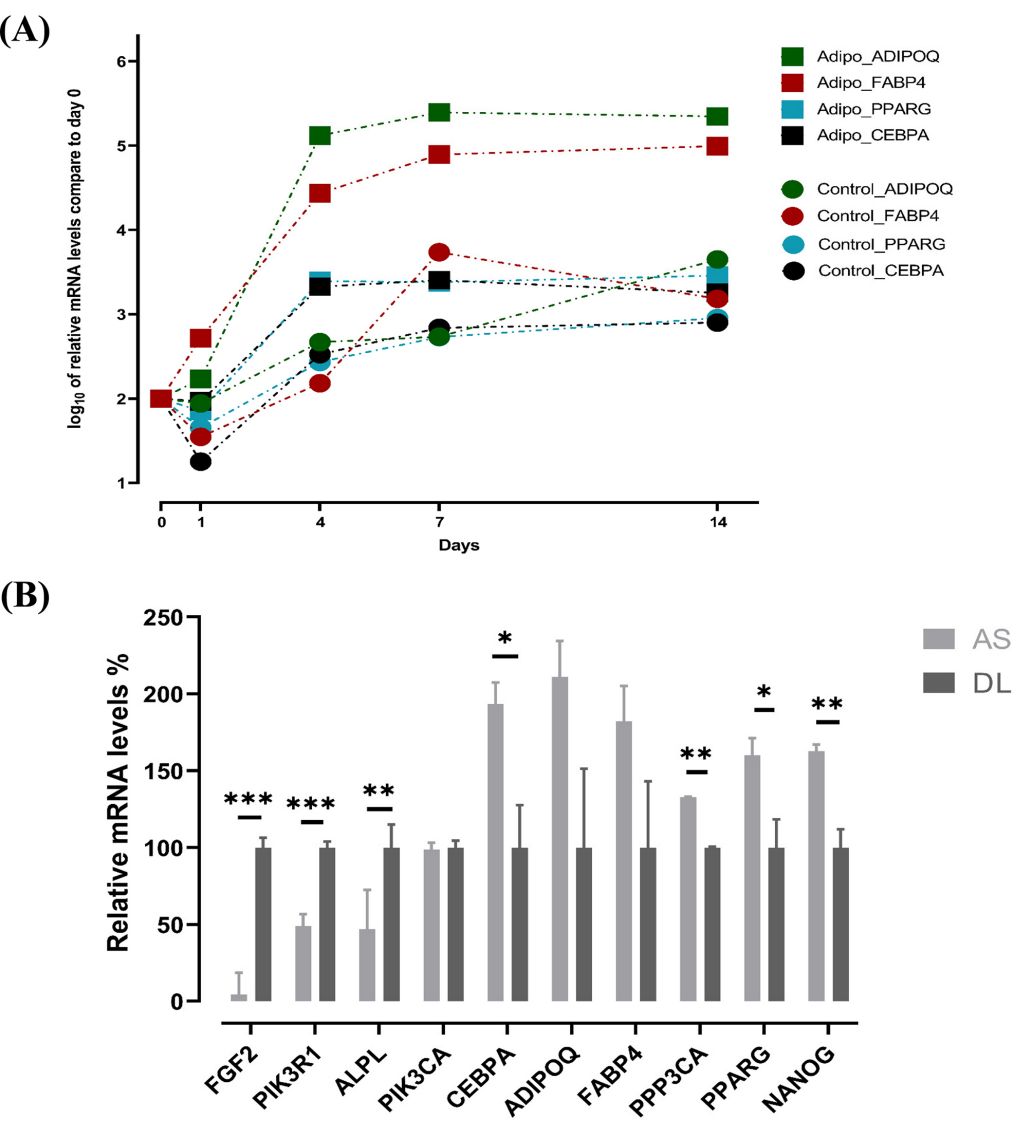
qRT-PCR validation of adipogenic marker transcripts. Expression of adipogenic marker genes during adipogenesis at each time point compared with control groups at day 0 by qRT-PCR (**A**). On day 1, no significant change was observed between the control and adipo groups. On day 4, there were significant differences between control and adipo groups: *CEBPA* (*P* = 0.002), *ADIPOQ* (*P* = 0.056) and *PPARG* (*P* < 0.0001). At day 7 and 14, all adipogenic genes were significantly different at *p* < 0.001, except *CEBPA* at day 14 (*p* = 0.07). Differential expression of synovial mesenchymal stem cells (SMSCs) transcripts derived from German Landrace (DL) and Angeln Saddleback (AS) confirmed by qRT-PCR (**B**). Least squares means and standard errors were used and then transformed to relative expression levels. *P < 0.05, **P < 0.01 and ***P < 0.001

We selected some PI3K-Akt signaling pathway genes (*PIK3R*1 and *PIK3CA*), adipogenesis markers (*CEBPA, ADIPOQ, FABP4*, and *PPARG*) and other genes of interest (*FGF2, NANOG, ALPL* and *PPP3CA*) for validation (Additional file 5). All of these adipogenesis markers were significantly different between the control and adipocyte-inducing groups. In particular, *ADIPOQ* and *FABP4* were significantly higher on day 7 in the adipocyte-induced group than in the control group, with fold change (FC) of 26.8 and 21.1, respectively, and on day 14, with FC of 23.6 and 28.2, respectively (Additional file 4; Fig. 7A). The Pearson correlation coefficient (r) ranged from 0.5 to 0.8 with a *p value* < 0.001 when comparing microarray and qRT-PCR data (*n* = 72).

As shown in Fig. 7B the expression of *FGF2, PIK3R1* and *ALPL* by RT-qPCR showed highly significant in DL breeds whereas some adipogenesis markers including *CEBPA* and *PPARG* were significant higher express in AS breeds. For *ADIPOQ* and *FABP4* showed tendency higher expression in AS particular on day 7 when consider the interaction of breed*stage (Additional file 5). *NANOG* and *PPP3CA* also significantly higher in SMSCs from AS than DL breeds.

### Longitudinal change of transcripts during adipogenesis

**Fig. 8:**
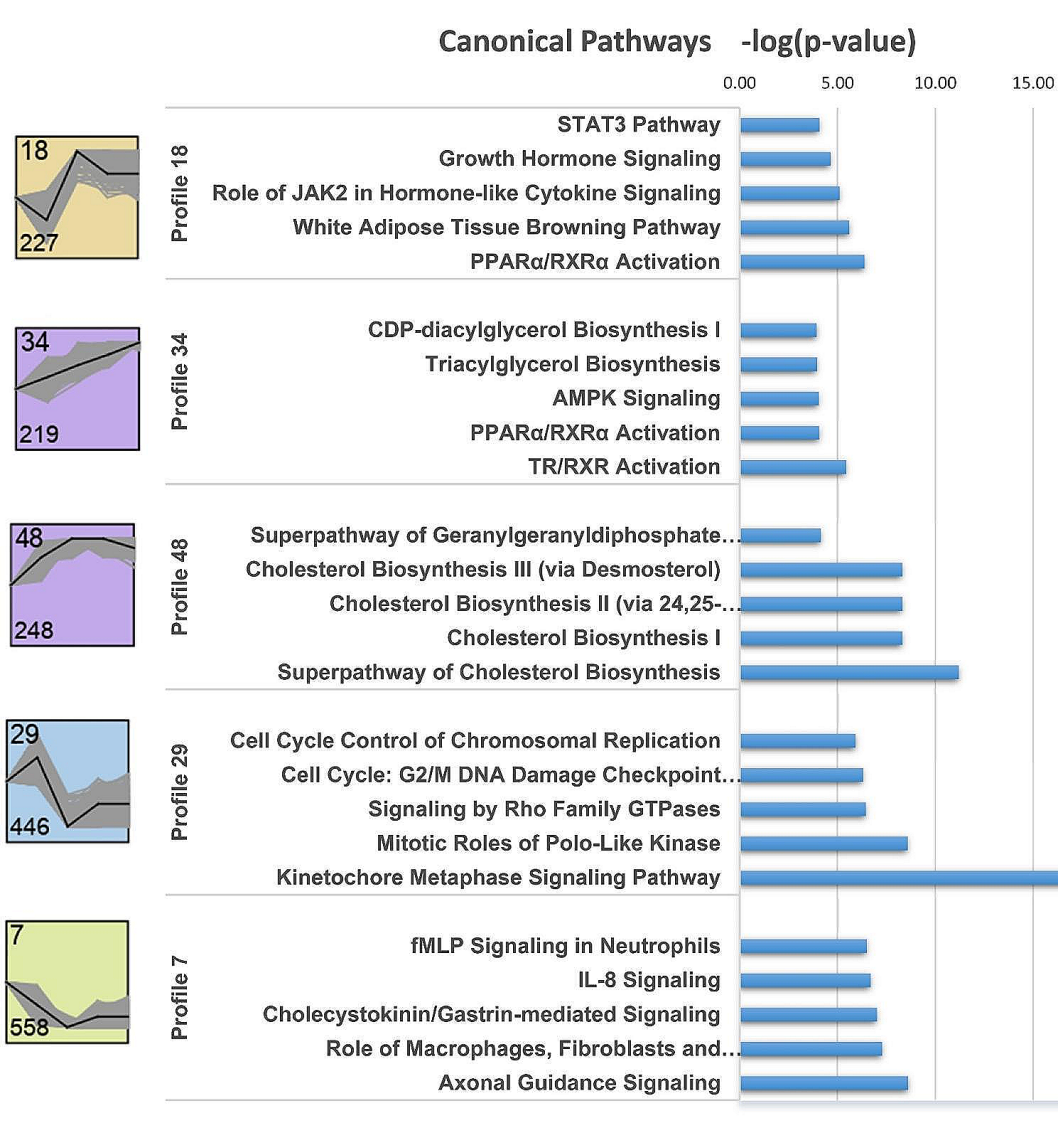
Longitudinal change profiles of transcripts during adipogenesis. Top 5 significant STEM profile and canonical pathways enriched by IPA. Profile 18, 34 and 48 display constantly increasing and profile 29 and 7 display constantly decreasing mRNA abundances during adipogenesis. The profile number was displayed at the top left and the number of genes at the bottom left

Short Time-series Expression Miner (STEM) analysis revealed statistically significant temporal expression profiles and the genes further used for functional enrichment analyses for groups of genes with the same temporal expression pattern. The 5 most significant profiles predominantly showed an upward (18, 34 and 48) or downward (29 and 7) trend in transcript abundances during adipogenesis (Fig. 8). Profile 18 with 227 transcripts with an initial decline in expression level from day 0 to day 1, an increase to day 7 and subsequent high level expression was enriched in immune pathways (STAT3 and JAK2 in hormone-like cytokine signaling), growth hormone signaling, white adipose tissue browning pathways and PPAR/RXR activation. Profile 29 included 446 transcripts with a reverse temporal expression pattern enriched in the cell cycle, particularly in the kinetochore metaphase signaling pathway. A total of 219 transcripts of profile 34 showed constant upregulation at each time point from day 1 to day 14 and were associated with triacylglycerol biosynthesis, AMPK signaling, PPARα/RXRα and TR/RXR activation. Transcripts related to cholesterol biosynthesis showed an increase in expression until reaching a plateau at day 7 and a smooth decline until day 14. A large set of 558 transcripts showed a decline in expression from day 0 to day 7 and consistently low further expression. They were associated with different signaling pathways, including axonal guidance or IL-8 signaling.

## Discussion

Adipogenesis, a developmental process to form fat tissue as energy storage tissue in which excess energy is stored in the form of lipids in adipocytes, is controlled by a complex cascade of adipocyte-specific transcription factors such as peroxisome proliferator-activated receptor γ (Pparg), CCAAT/enhancer-binding protein α (Cebpa), perilipin 2 (Plin2), fatty acid synthase (Fasn) and sterol regulatory element binding transcription protein 1 (Srebp1) [27, 28]. Furthermore, the specific regulation of cellular interactions in conjunction with many differentiation factors and extracellular matrix components are essential for the adipogenic differentiation process, such as signaling pathways, adhesion molecules and transcription factors [23, 29, 30]. During the early stages of adipogenesis, preadipocytes undergo clonal expansion and commitment to the adipogenic lineage (day1- day4). At this stage, the accumulation of intracellular lipid droplets is minimal and cells are still actively proliferating, as observed in our study with numerous signaling pathways related to the cell cycle, cell division, cell migration, or cell proliferation including *CEBPB*. As the differentiation process progresses, cells begin to accumulate intracellular lipid droplets and changes in gene expression patterns occur, such as increased expression of adipocyte-specific markers including *ADIPOQ*, *FABP4*, *PPARG* and *CEBPA*.

In this study, it was observed that PI3K-Akt signaling and metabolic pathways underwent the most significant changes during adipogenesis (AIM groups) compared to the control group (CCM groups). The PI3K/AKT signaling pathway plays a crucial role in regulating various cellular functions such as metabolism, growth, proliferation, survival, transcription and protein synthesis. It is particularly important for metabolic homeostasis and its function in insulin-sensitive tissues controlling glucose and lipid metabolism [31]. Knockdown of Akt1 by RNAi can inhibit the differentiation of 3T3-L1 preadipocytes [32], whereas activated Akt can promote the differentiation of 3T3-L1 preadipocytes into mature adipocytes [33]. Metabolic pathway alterations were observed throughout different stages of adipogenesis in this study. Notably, there was a significant upregulation of transcripts in the adipogenesis-induced group, with the most pronounced changes observed on day 7 and reaching their peak on day 14. These changes encompassed several key pathways, including PPAR signaling, AMPK signaling, fatty acid metabolism, regulation of lipolysis in adipocytes, unsaturated fatty acid biosynthesis, glycerolipid metabolism, adipocytokine signaling, peroxisome function, steroid biosynthesis, and pyruvate metabolism.

In this study, changes in transcripts associated with p53 signaling pathways and ferroptosis were identified during the late stage of adipogenesis, particularly on day 14. The role of p53 on adipocyte development, function and maintenance in white and brown adipose tissue was derived from in vivo and in vitro studies [34–36]. Ferroptosis, a form of programmed cell death dependent on iron-dependent lipid peroxidation, represents a novel aspect in adipogenesis research that remains poorly understood [37]. Notably, from day 4 to day 14, key regulator of ferroptosis, Glutathione Peroxidase 4 (*GPX4*), exhibited significantly upregulated transcripts in adipocytes, indicating a substantial difference compared to the control group.

A few transcripts exhibited differences when comparing SMSCs derived from FP or FS. Notably, specific biological processes, such as sterol biosynthesis and beta-oxidation of fatty acids, occur at a late stage in SMSCs derived from FP compared to FS. Interestingly, the expression of CCAAT/enhancer binding protein alpha (C/EBPα), a key transcriptional activator of adipocyte genes during adipogenesis, was significantly higher in FP than in FS. Consistent with findings from other studies, MSCs sourced from the anterior fat pad displayed a greater potential for adipogenic differentiation compared to MSCs from the surrounding synovium [38–40].

DL pigs are more muscular and less fat than AS pigs, which have a higher body fat content with larger fat deposits and intramuscular fat. During adipogenesis of AS-derived SMSCs compared to DL-derived SMSCs, different changes in biological and metabolic processes were observed. These different metabolic processes occur during the early and late phases of adipogenesis. We found that upregulated transcripts associated with metabolic pathways such as fat, energy or amino acid metabolism were particularly prominent on day 7 of adipogenesis in the AS breed. While the upregulated transcripts of SMSCs derived from DL were more enriched in immune signaling pathways on the same day. This suggests a shift in the biological and metabolic process of adipogenesis of SMSCs derived from AS and DL. The shift in metabolism in SMSCs from different genetic backgrounds, especially in relation to body fat, is an important clue and criterion for the selection of stem cell donors. The identification and analysis of specific genes, especially those linked to metabolic pathways in AS pigs, as well as pathways such as calcium signaling, immune signaling pathways or Wnt signaling in DL pigs, are crucial. Concentrating on these particular genes within their respective categories will significantly enhance our comprehension of adipose tissue development in diverse breeds, leading to advancements in animal husbandry practices within the field. Previous study showed mouse fed high fat diet alter bone marrow mesenchymal stem cells (BM-MSC) stemness and differentiation [41]. Increase evidence has indicated that overweight/obesity donor cell can change the bone marrow microenvironment, which affects some properties of bone marrow-derived mesenchymal stem cells (BMSCs) [42] or leads to undesirable proliferation, differentiation, and self-renewal of BMSCs [21]. In addition, obese-derived MSCs have lower potency compared to lean-derived MSCs, making them functionally impaired [43]. Donor genetic backgrounds contribute to the functional heterogeneity of stem cells and clinical outcomes particular for regenerative medicine and therapy was reported [44]. The presence of genetic and phenotypic variations in donor cells has the potential to limit the applications of induced pluripotent stem cells (iPSCs). It is intriguing to investigate whether diverse genetic backgrounds influence adipogenesis, particularly regarding the molecular phenotype.

Not surprisingly, the transcripts that show a temporal expression pattern with increasing abundance during adipogenesis from day 1 to day 14 belong to growth hormone signaling pathways, white adipose tissue browning, AMPK signaling pathways, triacylglycerol biosynthesis and PPAR/RXR activation. Metabolic pathways of interest such as white adipose tissue browning and triacylglycerol biosynthesis belong to the storage processes of excess dietary energy in the form of triacylglycerol lipid droplets in white adipose tissue (WAT). The phenomenon known as WAT browning or white-to-brown transition involves the accumulation of fat droplets rich in mitochondrial activity, leading to heightened oxygen consumption and heat generation [45]. Numerous transcripts involved in metabolic pathways, including *CEBPB, CREB3L2, FGFR1, FGFRL1, ITPR2, PLIN1, PRKAA2, PRKAR2B*, and *THRB* (associated with white adipose tissue browning), as well as *ABHD5, ELOVL6, GPAM, LPCAT3, CDS2*, and *LPIN1* (related to di/triacylglycerol biosynthesis), exhibited upregulation during the adipogenesis process in this study. Knowledge regarding changes in lipid composition during adipogenesis in SMSC is currently limited. However, a study conducted with human MSC revealed that adipogenic differentiation did not alter phospholipid composition or cholesterol content [46]. PPARg is known to regulate cholesterol metabolism in adipocytes [47]. This study observed an increase in the expression of several genes associated with the cholesterol biosynthesis pathways, reaching a plateau around day 7 and maintaining high expression. This temporal expression pattern aligns with that of PPARγ. Transcripts involved in cholesterol biosynthesis, such as *ACAT2, CYP51A1, EBP, FDFT1, FDPS, HMGCS1, HSD17B7, MSMO1, MVK, SC5D*, and *SQLE*, exhibited an increase during adipogenesis (profile 48). The PPAR pathway, known for promoting adipocyte differentiation and enhancing glucose uptake in the blood, is expressed in the late phase of differentiation when non-differentiated MSCs transition into pre-adipocytes. Similarly, the AMPK pathway, associated with adipocyte differentiation, follows a similar expression pattern [48–50].

In the early phase of differentiation, environmental signals control the differentiation potential and lineage establishment of MSCs [51]. Consequently, pathways associated with stem cell differentiation and cell division, including structures such as centrosome, kinetochores, and central spindle, were activated (profile 29). During the late stage of adipogenic differentiation, pathways involved in the regulation of lipid metabolism and inflammation were prominent. This aligns with previous studies indicating higher expression of genes related to adipocyte metabolism and lipid metabolism in MSCs undergoing adipogenic differentiation [52].

## Conclusions

Overall, adipogenesis is a complex and tightly regulated process involving the coordinated activation of transcription factors and signaling pathways. Genetic backgrounds that differ in metabolic phenotype play an important role in adipogenesis. Notably, the upregulation of molecular pathways related to fat, energy, or amino acid metabolism as early as on the day 7 of the differentiation process in AS compared to DL, highlights a distinct biological and metabolic shift in adipogenesis. This suggests a faster adipogenic differentiation potential in SMSCs from AS compared to DL. Differences in expression along differentiation depending on tissue suggest that anterior fat pads (FP) have a greater adipogenic differentiation potential than MSCs from the surrounding synovium (FS). Understanding the molecular mechanisms underlie adipogenesis is crucial for the development of therapeutic approaches to effectively treat obesity and associated metabolic disorders.

## Methods

### Cell isolation and differentiation experiments

We used cell samples from our previous study [12]. Briefly, three 59-day-old male piglets each of the breeds German Landrace (DL, *n* = 3) and Angler Saddleback (AS, *n* = 3) from the pig breeding unit of the Research Institute for Farm Animal Biology (FBN, Dummerstorf, Germany) were used. The pigs had ad libitum access to feed (Trede and von Pein, Itzehoe, Germany) and water in standard housing at the FBN Experimental Station. Pig were killed at the FBN slaughterhouse using electro stunning. The joint capsules of three male pigs each of the DL and AS breeds were aseptically opened under a laminar flow hood to obtain fibrous synovium (FS) from the inner side of the lateral joint capsule and adipose synovium (FP) from the inner side of the infrapatellar fat pad. Cells isolated on this occasion were used in our previous study and in this study after cryoconservation [12]. The detail of isolation methods, culture conditions, and morphological aspects and molecular characterization of synovial mesenchymal stem cells (SMSCs) derived FS and FP was reported in our previous study [12]. These SMSCs were confirmed to express surface marker profiles indicative of stemness by positive immunofluorescence staining and flow cytometry for CD44, CD29, CD90, and CD105, as reported in our previous studies [12]. We pooled the cells in passage 3 from three donor animals of each breed and each tissue and used these pools in two replicates each at all-time points throughout the study. SMSCs at passage 3 were trypsinized, re-seeded to 2 × 10^4^ cells/well in a 24 well plate with complete culture medium (CCM) (HG-DMEM, 4,500 mg/L glucose Dulbecco’s modified Eagle’s medium, Gibco, New York, USA), supplemented with 10% FBS (Sigma-Aldrich, St Louis, USA) and 1% antibiotic/antimycotic solution. For specific differentiating conditions, adipogenic induction medium (AIM) (StemPro™ adipogenesis differentiation kit, Thermofisher) was used. Adipogenic differentiation was induced by culturing the cells for up to 14 days. The FP- and FS-derived cells from DL and AS in AIM medium were each grown in two independent cultures and collected on day 1, 4, 7 and 14. In addition, non-differentiated cells cultured in CCM medium were kept in parallel as a control group and collected at the beginning of the experiments (day 0) and subsequently at the same time points as the differentiation group (1, 4, 7 and 14 days). A total of 72 samples (differentiated cells (AIM groups): collected at 4 time points × 2 tissues × 2 breeds × 2 replicates) + (non-differentiated cells (CCM groups): collected at 5 time points × 2 tissues × 2 breeds × 2 replicates) were used.

### Histochemistry

To control for adipogenic differentiation, the cells were gently rinsed three times with phosphate-buffered saline (PBS). Subsequently, the cells were fixed with a 4% formalin solution (in PBS) for 30 min at room temperature (RT) and then carefully washed twice with deionized water (ddH2O). The cells were stained with Oil Red O Staining Solution, which contained 60% isopropanol and 1 mg/ml of Oil Red O (Sigma-Aldrich, Taufkirchen, Germany), for 15 min at RT. Following the staining procedure, excess stain was removed, and all stained cells were examined microscopically and photographed using a Nikon Microphot-FXA/FXL microscope.

### RNA isolation, target preparation and hybridization

Total RNA of 72 samples describe above was extracted with TRI reagent (Sigma-Aldrich, Taufkirchen, Germany) and RNeasy kit (Qiagen, Hilden, Germany). Then, they were cleaned and purified with DNase and a column-based NucleoSpin RNA II-Kit (Macherey-Nagel, Düren, Germany). The quality and quantity of isolated RNA were checked by NanoDrop ND-1000 spectrophotometer (Peqlab, Erlangen, Germany), checked for integrity by performing agarose gel electrophoresis (1% agarose gel), and the extracted RNA were stored at -80 °C for further analysis. To prepare the samples for microarray analysis, 500 ng RNA was used for generating amplified sense-strand cDNA using the Affymetrix GeneChip WT PLUS Reagent Kit (Affymetrix, Santa Clara, CA, USA). Next, cDNA was fragmented and biotin-labeled using the Affymetrix GeneChip WT Terminal Labeling Kit (Affymetrix, Santa Clara, CA, USA). Each individual sample was hybridized on a genome-wide snowball array (Affymetrix, Santa Clara, CA, USA), containing 47,880 probe-sets. After staining and washing, Affymetrix GCOS 1.1.1 software was used for scanning and processing the arrays data.

### Microarray data processing, statistical and pathway analyses

The snowball Microarray (Affymetrix) with 47,880 probe sets representing 17,964 annotated genes was used to determine the expression profile. Analysis of the microarray data was performed using the Expression Console 1.3.1.187 software platform (Affymetrix) to translate intensities of chip-spots into values for the expression of transcripts represented by probe-sets as described in our previous study [53]. Background correction, normalization, and summarization was performed using the Robust Multiarray Average (RMA) algorithm. The DABG (Detection Above Background) algorithm was used to filter present and absent genes. Probe sets present in less than 80% of the total number of samples were excluded for further analysis. In addition, probe sets with expression values with a standard deviation of less than or equal to 0.25 (≤ 0.25) across all experimental conditions were filtered out to reduce the number of hypotheses to be considered in the multiple test adjustment. Finally, a total of 13,286 probe sets were used for subsequent analyses.

Linear model procedure available under Row-by-Row Modelling sub-menu from JMP genomics 9.0 software (SAS Institute, Cary, NC, USA) was used to analyze the differential gene expression between tissues, breeds, and state of SMSCs (adipocyte differentiation or control group at each collection day) and their interactions with breeds and tissues. The adjusting for multiple comparisons for all fix effects (tissues, breeds, and state of SMSCs) were calculated by using the post hoc Tukey-Kramer test. The repeated statement of the Proc MIXED was used to consider and take advantage of the repeated measurements of each of the pools at all-time points. Probe sets with expression values differing between groups at an adjusted *p*-value (FDR) of less than 0.01 represent transcript entities with significantly different abundance and were registered as differentially expressed genes (DEGs). The microarray data were then deposited in a public database GSE232501: GSM7349177-GSM7349248.

### Real-time quantitative PCR (qPCR) validation

DEGs were selected for qPCR validation using a 48 × 48 Dynamic array with an integrated fluidic circuit (IFC) on the BioMark HD Real-time PCR System (Fluidigm, South San Francisco, CA, USA). Specific target amplification (STA) was performed according to the manufacturer’s recommendations and according to our previous study [53]. Pre-amplification sample mixtures were prepared using PreAmp Master Mix (Fluidigm PN 1,005,581) containing 1.25 µL of cDNA, 1 µL PreAmp Master Mix, and 0.5 µL Pooled Delta Gene Assay Mix (500 nM) containing DNA-suspensions buffer and primers mixes in 5 µL total volume. The preamplification reaction was incubated at 95 °C for 2 min, followed by 10 cycles at 95 °C for 15 s and 60 °C for 4 min. Fluidigm quantitative measurement runs were carried out with 48.48 dynamic arrays (Fluidigm Corporation, CA, USA) according to manufactures instructions. In brief, 2.5 µL of 2 × SsoFast Evagreen Supermix with Low ROX, 0.25 µL 20 × sample-loading reagent, and 2.25 µL of treated samples were prepared. Separately, an assay mixture was prepared for each primer pair and this included 2.25 µL of DNA Suspension buffer, 0.25 µL of 100 µM forward and reverse primer, and 2.5 µl of 2× assay-loading reagent. The array chips were placed in the BioMark Instrument for PCR at 95 °C for 10 min, followed by 30 cycles at 95 °C for 15 s and 60 °C for 1 min. The data were analyzed using software in the BioMark HD instrument (Fluidigm Corporation, San Francisco, CA). The internal controls of 3 housekeeping genes including *HPRT1, PPIA* and *YWHAZ* were used. Pearson correlation coefficient (r) between the microarray and qPCR data was performed using SAS version 9.4 (SAS Institute). Ten genes were selected for validation and the primers sequence was showed in Additional file 5.

### Electronic supplementary material

Below is the link to the electronic supplementary material.

Supplementary Material 1

Supplementary Material 2

Supplementary Material 3

Supplementary Material 4

Supplementary Material 5

## Data Availability

The expression datasets for this study can be found in the Gene Expression Omnibus public repository with the GEO accession number (GSE232501: GSM7349177-GSM7349248).
